# In-vivo evaluation of a reinforced ovine biologic: a comparative study to available hernia mesh repair materials

**DOI:** 10.1007/s10029-019-02119-z

**Published:** 2020-01-31

**Authors:** N. Overbeck, G. M. Nagvajara, S. Ferzoco, B. C. H. May, A. Beierschmitt, S. Qi

**Affiliations:** 1 TELA Bio, Inc., Malvern, PA USA; 2 Department of Surgery, Atrius Health, Dedham, MA USA; 3 Aroa Biosurgery Limited, Auckland, New Zealand; 4 Behavioural Science Foundation, Basseterre, Saint Kitts and Nevis; 5 University of Montreal, Montreal, QC Canada

**Keywords:** Hernia repair, Mesh repair materials, Biologic, Synthetic, Decellularized extracellular matrix

## Abstract

**Purpose:**

Two innovative reinforced biologic materials were studied in a non-human primate hernia repair model. The test articles, which combine layers of ovine decellularized extracellular matrix with minimal amounts of synthetic polymer, were evaluated for their biologic performance as measured by inflammatory response, healing kinetics, integration, and remodeling into functional host tissue. For comparison, seven clinically used biologic and synthetic meshes were also studied.

**Methods:**

Animals were implanted with test articles in surgically created full-thickness midline abdominal wall defects, and evaluated macroscopically and histologically at 4, 12, and 24 weeks.

**Results:**

Macroscopically, biologics resorbed and remodeled into naturally appearing tissue; the reinforced biologics appeared similar, but remodeled earlier and were less prone to stretch. Synthetics developed a layer of reactive tissue above and separate from the contracted mesh structure. At early time points, the collagen networks of biologics and reinforced biologics were infiltrated by host cells primarily as a peripheral layer on the biologics. As early as 12 weeks, the collagen networks associated with the reinforced biologics remodeled into organized host collagen. By 24 weeks, both reinforced biologics and biologics had low levels of inflammation. In contrast, a foreign body response persisted at 24 weeks with the synthetics, which had developed less organized collagen, separate in space from the actual mesh.

**Conclusions:**

The current study shows a favorable response to reinforced biologics, which were associated with an initial inflammatory response, resolving by later time points, followed by active remodeling, and the formation of new morphologically functional collagen.

**Electronic supplementary material:**

The online version of this article (10.1007/s10029-019-02119-z) contains supplementary material, which is available to authorized users.

## Introduction

Hernia repairs represent one of the most common surgical procedures performed. The introduction of reinforcement of incisional or ventral hernia repair with synthetic meshes has significantly reduced the risk of recurrence, i.e., post-operative failure of the hernia repair. Despite that, recurrence remains a common problem. A landmark randomized prospective study found a 10-year cumulative incidence of recurrence of 63% for suture repairs and 32% for mesh reinforced suture repairs [1]. However, data from the Danish registry study showed that the benefits of synthetic mesh reinforced repairs diminish over time, as on average about 1% of patients each year undergo an additional surgical procedure for mesh related complications [2].

Traditional meshes have typically been classified into three broad classes: permanent synthetics, resorbable synthetics, and biologics. The most widely used meshes are permanent synthetics; made either from polypropylene (PP), polytetrafluoroethylene (PTFE), or polyester. Although these materials are strong and affordable, their usage is associated with dose-dependent chronic inflammation, scar formation, migration, and nerve entrapment [3]. Additionally, these meshes become brittle and contract over time, and are difficult and destructive to remove [4]. Resorbable synthetics, made either of combinations of polyglycolic acid (PGA), polylactic acid (PLA), and trimethylene carbonate (TMC), or poly-4-hydroxybutyrate (P4HB) have similar attributes to permanent synthetic meshes once implanted but have a relatively short clinical history. As such, there are limited data on recurrence rates after the implants are fully resorbed. In one such study, implants made from 67% PGA and 33% TMC that resorbed in 6 months had a 17% recurrence rate at 2 years [5]. A prospective multicenter study for P4HB reported 19 recurrences in 82 patients, or 23%, at 3 years [6].

Biologic materials have gained favor for repair of hernias in infected or contaminated fields and other high-risk patients [7]. Biologics are produced from mammalian (e.g., human, ovine, bovine, or porcine) source tissues that undergo processes to remove the cellular components, leaving an intact and functional tissue extracellular matrix (ECM). ECMs that are properly decellularized (dECM) retain signaling and adhesion molecules that promote the proliferation of fibroblasts, deposition of collagen, and the development of new blood vessels, and provide a three-dimensional scaffold for the growth and remodeling into native tissue at the defect site [8, 9].

Although biologics provide support and a scaffold for tissue-specific cell growth, signaling, and remodeling, they are expensive, and, more importantly, clinical evidence shows the remodeled tissue may stretch over time [10, 11]. Furthermore, the very fact that biologics are derived from different human or animal tissues, such as dermis, small intestinal or bladder submucosa, and pericardium—contributes to significant differences in performance. This is further compounded by differences in the decellularization processes used and other manufacturing steps [12]. Based on the characteristics and clinical results described, there clearly is room for improvement in hernia repair materials.

An improved surgical mesh material for soft-tissue reconstruction would combine desirable features of both biologic and synthetic materials resulting in a reduction and attenuation of the inflammatory effect of the implant, resistance to infection, rapid remodeling into functional tissue, and the prevention of overextension and laxity.

Reinforced biologics were developed to provide such an improved construct. The reinforced biologics combine the adventitious properties of biologics and synthetics, and consist of layers of intact and functional dECM, namely ovine forestomach matrix (OFM), embroidered together with minimal amounts of synthetic polymer to specifically reinforce the construct. They have been used clinically since July 2016, and at the time of this writing, they have been implanted in over 7000 patients. The biological response and integration following implantation of the reinforced biologics was evaluated in a non-human primate model and directly compared to clinically used synthetic and biologic devices. The objective of the study was to evaluate the overall healing of the defect as evidenced by the cellular response, and the morphological appearance of the tissue associated with each implant. Although this model had some limitations in terms of mimicking the clinical aspects of the condition and its repair, it served to provide comparative and relevant data in a large animal model through a 24-week period of time.

## Materials and methods

### Test articles

Nine test articles were evaluated in this study, including the two novel reinforced biologics, OviTex 1S^®^ Resorbable and OviTex 1S^®^ Permanent (designed by TELA Bio, Malvern, PA, USA), as well as Phasix™ (C.R. Bard, Inc./Davol, Inc., Warwick, RI, USA), Ventralight ST™ (C.R. Bard, Inc./Davol, Inc., Warwick, RI, USA), Physiomesh™ (Ethicon, Inc., Somerville, NJ, USA), Strattice™ Firm [LifeCell Corporation, Branchburg, NJ, USA (now Allergan)], SurgiMend 1.0^®^ (Integra LifeSciences Corp., Plainsboro, NJ, USA), Gentrix^®^ Surgical Matrix Plus (ACell Inc., Columbia, Maryland), and Zenapro^®^ (Cook Medical, West Lafayette, IN, USA) (Table 1). Materials were prepared according to their respective IFUs.

**Table 1:** Test articles, mesh classification, source materials, and explant time points

Mesh	Manufacturer	Class	Composition	Time point (weeks)	Animals per time point
OviTex^®^ PGA 1S	Developed, designed and manufactured by: Aroa Biosurgery Limited and TELA Bio, Inc	Reinforced biologic	Decellularized ovine forestomach matrix ECM embroidered with polyglycolic acid (PGA)	4, 12, 24	3
OviTex^®^ PP 1S		Reinforced biologic	Decellularized ovine forestomach matrix ECM embroidered with polypropylene (PP)	4, 12, 24	3
Phasix™	CR Bard (Davol)	Resorbable synthetic	Poly-4-hydroxybutyrate (P4HB) and polyglycolic acid (PGA)	4, 12, 24	3
Ventralight ST™	CR Bard (Davol)	Permanent synthetic	Polypropylene and polyglycolic acid (PGA) with sodium hyaluronate (HA) carboxymethylcellulose (CMC) and polyethylene glycol (PEG) based hydrogel barrier	4, 12, 24	3
Physiomesh™^a^	Ethicon	Permanent synthetic	Polypropylene laminated between polyglecaprone-25 films bond w/polydioxanone film	4, 12, 24	2
Strattice™ Firm	LifeCell (now Allergan)	Biologic	Decellularized porcine dermal ECM	4, 12, 24	5
SurgiMend 1.0™	TEI Biosciences (now Integra)	Biologic	Decellularized fetal bovine dermal ECM	4, 12 weeks	3
Gentrix^®^ Surgical Matrix Plus	ACell	Biologic	Decellularized porcine urinary bladder ECM	4, 12	2
Zenapro™^a^	Cook Medical	Hybrid biologic	Decellularized porcine small intestinal submucosa ECM and polypropylene	4, 12	3
Total animals					73

^a^No longer in distribution

### Reinforced biologics

Reinforced biologics were prepared from layers of ovine forestomach matrix (OFM) produced from ovine (sheep) forestomach using proprietary decellularization methods (Aroa Biosurgery, New Zealand). OviTex 1S^®^ Resorbable and OviTex 1S^®^ Permanent were created from layers of OFM, using either polypropylene (PP) or polyglycolic acid (PGA) suture (2-0). All devices were terminally sterilized (ethylene oxide) prior to use in the study. Implants used in this study consisted of six layers—four layers of OFM, embroidered with a 6-mm grid pattern, and two additional OFM layers to prevent intestinal adhesion on one side using a 25-mm grid design.

### Surgical procedure

Seventy-three (73) adult vervet monkeys (*Cercopithecus aethiops*) (3–6 kg) obtained through the Behavioural Science Foundation (BSF), St. Kitts were used in this study. The protocol for this study was reviewed and approved by the BSF Institutional Animal Care and Use Committee (IACUC). BSF holds a certificate of Good Animal Practice with the Canadian Council on Animal Care (CCAC) and observes Guidelines for the Care and Use of Laboratory Animals (as outlined in NIH Publication #85-23 Rev. 1985). Animal screening and handling, as well as full-thickness 7 × 3-cm abdominal wall defect creation, implant sample inlay (“bridging”) repair, and post-surgical treatment procedures were performed as described in the literature [13, 14]. Study details are presented in Table 1.

The monkeys were screened for general health and quarantined for a minimum of 32 days prior to study entry. Two weeks or less, prior to study commencement, the animals were given complete physicals, observed for good health, and then moved to single-cage stainless steel housing where they can see other conspecifics and receive daily enrichment to encourage normal species-specific behaviors. The animals remained in this housing throughout the study to prevent normal cage mate behaviors from possibly damaging the implant site.

An initial intramuscular injection of Ketamine HCl (10 mg/kg) was used to handle the animals and bring them to surgery where they were weighed and aseptically prepped, and an IV catheter was placed in the saphenous vein. A maintenance rate of Lactated Ringers is given throughout surgery. A surgical plane of anesthesia was maintained with xylazine and ketamine, given by intramuscular injection (1:10, 10 mg/kg). A pre-op dose of a broad-spectrum antibiotic was used.

A longitudinal mid-abdominal skin incision of approximately 7 cm was made to expose an area of the linea alba and muscle wall. Then, a 7 × 3-cm full-thickness “window” defect was created in the midline of the abdominal wall removing sections of both rectus muscles, including the fasciae and the peritoneum.

The abdominal wall defect was then repaired with an implant of the test sample equal in size to the defect (approximately 7 × 3 cm) using an inlay (‘bridging’) approach (see Table 1 for number of animals per group). The implant was anchored at each of the four corners with 2-0 non-absorbable polypropylene sutures in an interrupted pattern, and was sutured to the edges of the rectus abdominal muscle and fascia with non-absorbable 2-0 polypropylene running sutures.

The subcutaneous tissue was closed with an absorbable polydioxanone suture (2-0) in a running pattern. Finally, the skin was closed with non-absorbable nylon sutures (2-0) in an interrupted pattern.

An intra operative dose of an opioid and an NSAID was given for analgesia and continued for a minimum of 3 days post op or as deemed necessary from pain scoring at observations.

Animal care technicians recorded clinical observations at least once daily for the duration of the study, and body weight was recorded at each physical examination. In addition, a daily pain scale was employed to ensure animals were provided adequate post-surgical pain relief and in the event of any painful procedure or event.

### Implant recovery

Implants were recovered at 4, 12, and 24 weeks (Table 1), and evaluated by a veterinarian for signs of herniation, inflammation, adhesions, contraction, or other abnormalities as well as evidence of healing and integration as described in the literature [13, 15, 16]. Adhesion tenacity was scored on strength from zero to three, where 0 = no adhesions, 1 = adhesions easily freed with gentle tension, 2 = adhesions freed with blunt dissection, and 3 = adhesions requiring sharp dissection to be freed [10]. The length and width of the implant was measured in-situ for calculation of percent of the starting area (21 cm^2^) as well as aspect ratio (determined as the ratio of in-situ implant length to width). The entire implant and surrounding tissue were removed and photographed. A midline cross section of each implant was removed, cut in half, and placed in 10% neutral buffered formalin for histologic analysis.

### Histology and histopathology

The formalin fixed specimens were embedded in paraffin, cut into sections, and stained with hematoxylin and eosin (Tejas Pathology Associates, Tomball, TX, USA). Two slides were prepared from each animal: left and right host–implant interface. The slides were evaluated by a pathologist who was blind to treatment and time point (CBSET Inc, Lexington, MA, USA). Established standard toxicological pathology criteria were used as a guide to create a scoring methodology categorizing the microscopic tissue appearance on a scale from 0 to 4 (Table 2). Values obtained from the histomorphology analysis were entered in Microsoft^®^ Excel^®^, reported as the group median, mean ± SD, and percent incidence. Calculations, data organization, and graphs were generated using Minitab^®^ software (Version 17). Inflammation was scored on the extent of inflammatory cells (macrophages, neutrophils, giant cells, lymphocytes, and plasma cells) within or associated with the test article. Pertinent microscopic observations were scored for intra- and peri-implant cellular responses, implant presence, fibrosis/collagen organization, and vascularization. Abdominal wall defect-associated tissue was scored comparing the thickness of the defect area to the abutting abdominal tissue. Implant-to-tissue ratio was quantified based on the amount of remaining implant material in relation to the total thickness of the tissue inside the defect site.

**Table 2:** Histomorphology scoring matrix

Score	Inflammation/inflammatory cells	Pertinent microscopic observations	Abdominal wall defect-associated tissue	Implant-to-tissue ratio
0	Absent	No response/not detectable	Absent	Absent
1	Rare, minimal 1–5/per high power field (hpf; 40 × obj)	Minimal/focal/barely detectable	Minimal (i.e., notably thinner)	< 25%
2	Mild, 5–10/hpf	Mild/focal or rare multifocal/slightly detectable	Mild (i.e., slightly thinner)	25–50%
3	Heavy infiltrate, with preservation of local architecture	Moderate/multifocal to confluent/easily detectable	Moderate (i.e., equivalent in thickness)	50–75%
4	Packed, with effacement of regional architecture	Marked/diffuse/overwhelming presence	Marked (i.e., thicker than the abdominal wall)	> 75%

## Results

### Gross necropsy observations

**Fig. 1:**
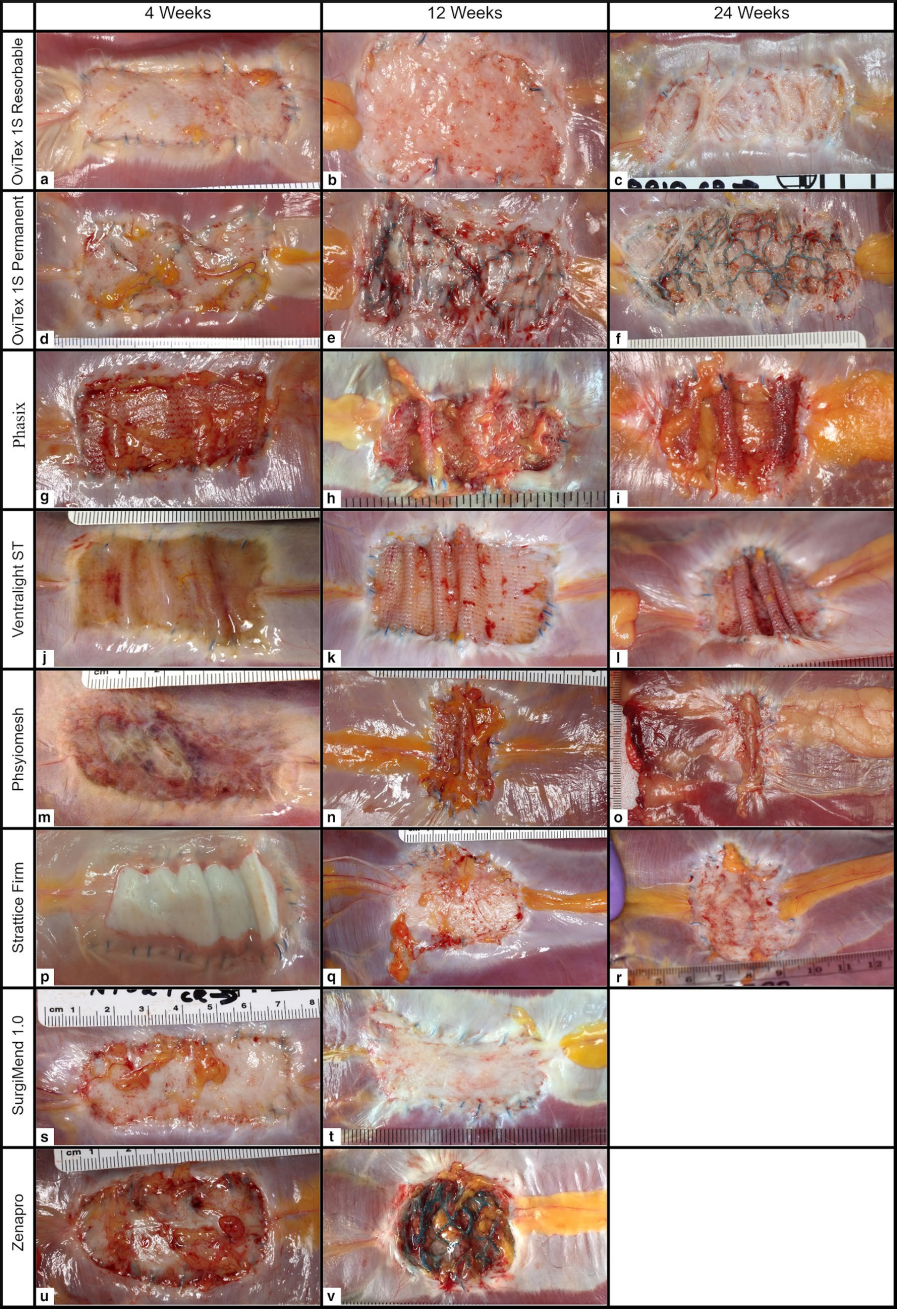
Gross necropsy images of implants at 4, 12, and 24 weeks

Representative images for each of the test samples at each time point are provided in Fig. 1.

There were no signs of herniations in any of the animals. However, the two Gentrix Surgical Matrix Plus implants at 4 weeks had torn away from approximately 75% and 50% of the defect perimeter, respectively, and those at 12 weeks could not be identified in the defect area (Supplementary Fig. 1). Based on this, Gentrix Surgical Matrix Plus was not included in any further analyses.

All animals showed surface adhesions of the omentum, which were freed with blunt dissection except for Phasix and Physiomesh, which required sharp dissection to remove at 24 weeks (Table 3).

**Table 3:** Average in-situ length, width, area, percent of starting area, aspect ratio, and omentum adhesion strength

Group	In situ length (cm)	In situ width (cm)	In situ area (cm^2^)	In situ percent starting area	In situ aspect ratio (L:W)	Omentum adhesion strength
4 weeks						
OviTex 1S Resorbable	5.7	2.2	13.1	0.6	2.6	1.7
OviTex 1S Permanent	5.1	2.7	13.7	0.7	1.9	2.3
Phasix	5.0	2.5	12.4	0.6	2.0	2.0
Ventralight ST	4.2	2.6	11.1	0.5	1.6	1.0
Physiomesh	5.6	3.0	16.3	0.8	2.0	1.5
Strattice Firm	4.9	2.9	14.2	0.7	1.7	2.0
SurgiMend 1.0	5.8	2.9	16.9	0.8	2.0	2.0
Zenapro	5.2	2.7	14.2	0.7	2.0	2.0
12 weeks						
OviTex 1S Resorbable	4.5	3.5	15.9	0.8	0.5	1.0
OviTex 1S Permanent	4.1	3.0	12.2	0.6	0.5	1.7
Phasix	4.2	2.7	11.1	0.5	0.6	2.0
Ventralight ST	4.3	2.3	10.1	0.5	0.1	1.0
Physiomesh	3.6	2.8	8.7	0.4	1.6	2.0
Strattice Firm	4.3	3.4	13.9	0.7	0.8	2.0
SurgiMend 1.0	4.4	3.0	14.1	0.7	0.9	1.3
Zenapro	4.0	3.1	12.2	0.6	0.0	2.0
24 weeks						
OviTex 1S Resorbable	5.2	2.5	13.6	0.6	0.6	1.7
OviTex 1S Permanent	6.7	2.4	16.3	0.8	0.7	2.0
Phasix	4.3	2.8	11.4	0.5	0.9	3.0
Ventralight ST	3.4	2.6	8.8	0.4	0.3	1.7
Physiomesh	1.9	3.9	7.2	0.3	0.0	3.0
Strattice Firm	4.5	5.4	23.2	1.1	1.0	1.4
SurgiMend 1.0	–	–	–	–	–	–
Zenapro	–	–	–	–	–	–

There was a wide variety of implant geometry at the various time points as the implants contracted or stretched to different degrees in length and width (Fig. 1). The geometry was analyzed by measuring the percent reduction in defect area, and the aspect ratio (measure of length over width) (Table 3). All implants at all time points showed a reduction in area (Table 3), except for Strattice Firm, which at 24 weeks had stretched to 110% of the original defect area. The most contracted implants were the synthetics, which had reduced to less than 50% of the defect area by 12 weeks and further reduced by 24 weeks, forming rolls in the mesh (Fig. 1i, l, o). At 4 weeks, test articles essentially retained their aspect ratios (approx. 2.33). However, by 24-week differences between the test articles were observed. For example, at 24 weeks, the reinforced biologics best preserved the original aspect ratio of the defect area (2.45 average), whereas Physiomesh was substantially distorted (0.45 average).

### Histology

**Fig. 2:**
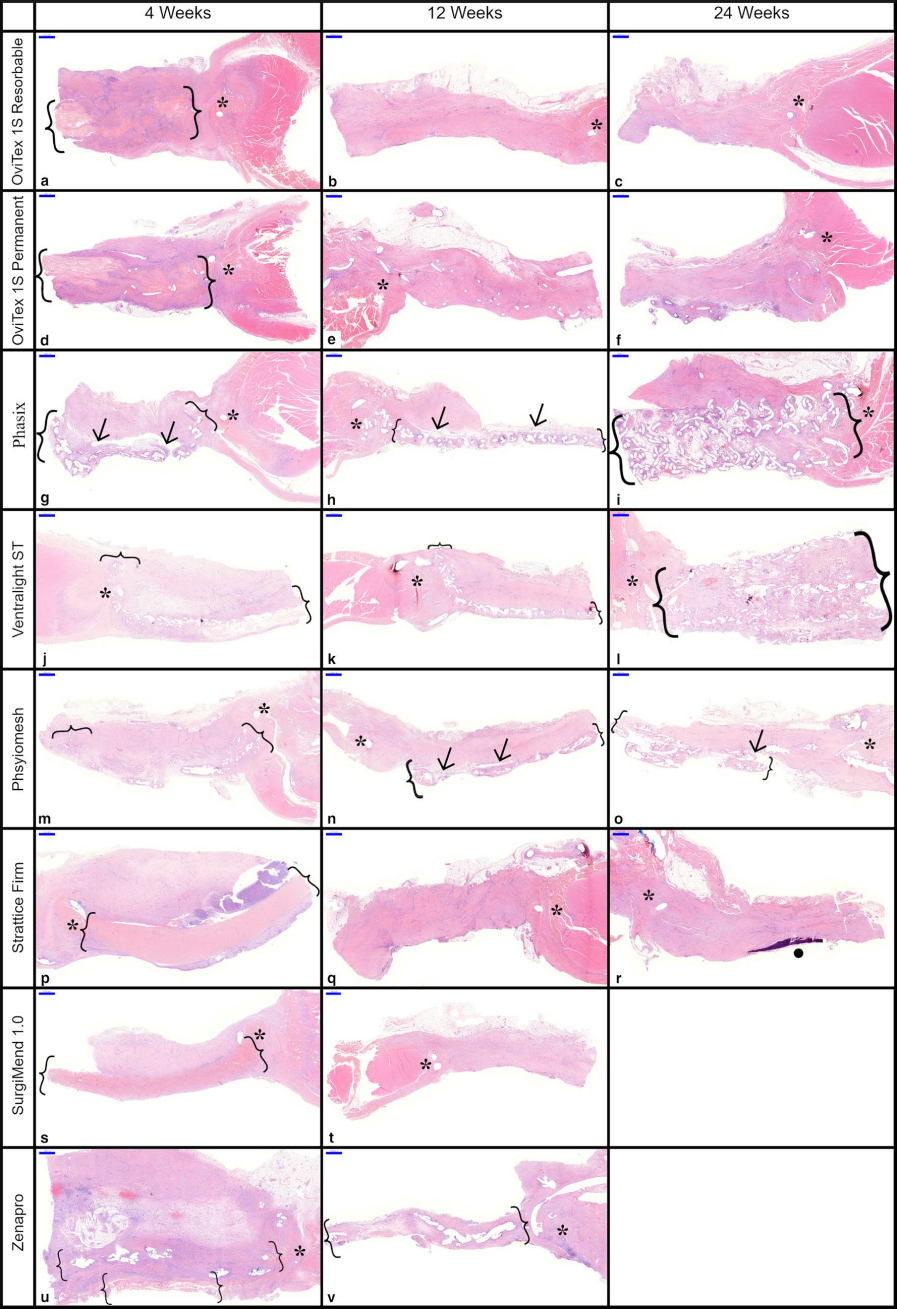
Low magnification histology of all implants at 4, 12, and 24 weeks. Brackets = implant material, asterisk = implant to ab wall anchoring suture, arrows = delamination, and dot = calcification/osseous metaplasia. Scale bar 1000 µm

**Fig. 3:**
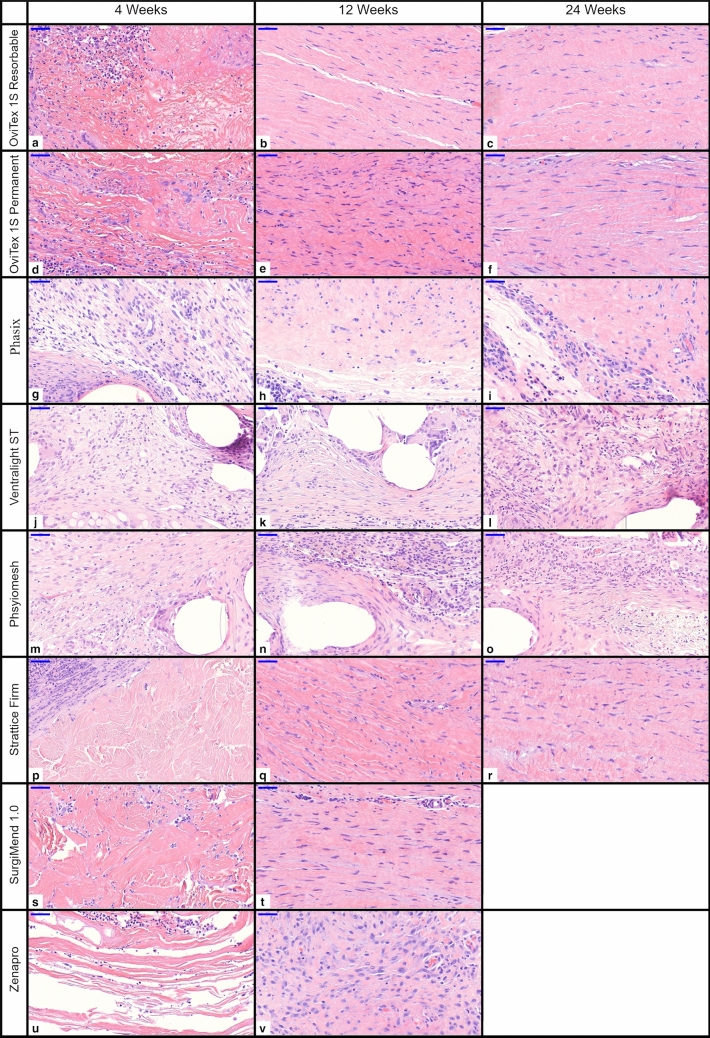
High magnification histology of all implants 4, 12, and 24 weeks, scale bar 50 µm

Representative low and high magnification histology images for each of the test samples at each time point are provided in Figs. 2, 3.

### Inflammation

**Fig. 4:**
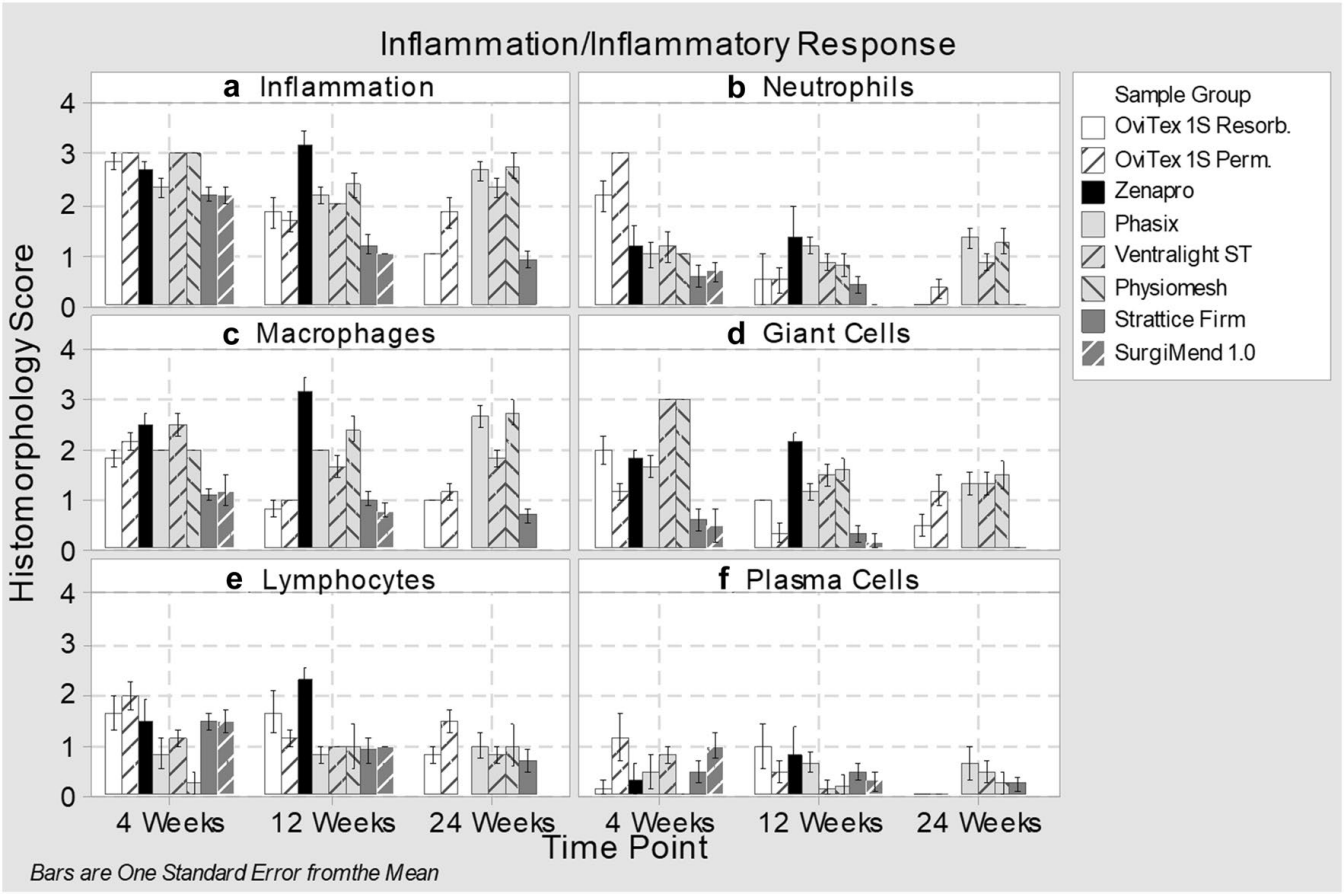
Histomorphological scoring for inflammation/inflammatory response to implants at 4, 12, and 24 weeks

Inflammation at 4 weeks was mild-to-moderate (score 2–3) across all groups (Fig. 4a). Among implants at 24 weeks, inflammation was minimal (less than 1) with OviTex 1S Resorbable and Strattice Firm, mild with OviTex 1S Permanent, and mild–moderate for the synthetic meshes Phasix, Physiomesh, and Ventralight ST.

Biologics and reinforced biologics were initially infiltrated primarily by lymphocytes and macrophages, and in the case of reinforced biologics, neutrophils (Fig. 4b–d). At 12 and 24 weeks, macrophage infiltrate decreased to near minimal and near absent, respectively, as the implants were integrated into host tissue. Synthetic devices were primarily infiltrated by histiocytic cells, with giant cells and macrophages in the areas immediately surrounding the synthetic materials. The inflammatory response to these devices persisted at elevated levels throughout the study (Fig. 4a).

### Host cellular infiltration and fibroproliferative remodeling: biologics and reinforced biologics

**Fig. 5:**
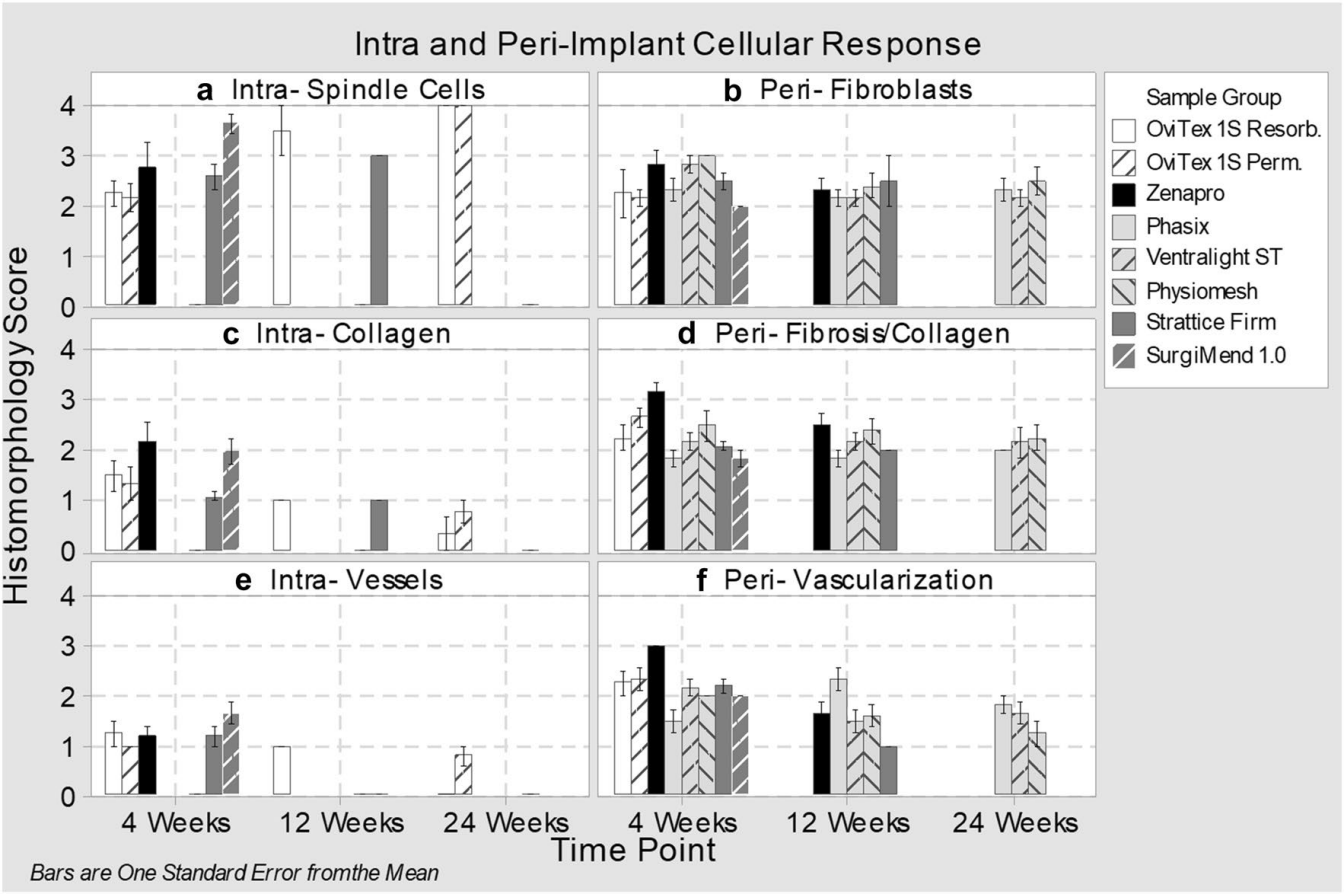
Histomorphological scoring for intra- and peri-implant cellular response to implants at 4, 12, and 24 weeks

Implants containing biologic materials were evaluated for the degree and timing by which they were infiltrated by host tissue such as fibroblasts and collagen, and for the formation of vasculature. At 4 weeks, infiltration by spindle cells was mild-to-moderate (score 2–3) with both OviTex implants, Strattice Firm and Zenapro, while extensive with SurgiMend 1.0 (Figs. 3a, d, p, s, 5a). Collagen deposition and blood vessel infiltration were minimal-to-mild with all groups (score 1–2). OviTex 1S Resorbable at 12 weeks and all other biologic containing implants had diffuse tissue infiltration throughout the interstitium between individual collagen bundles at 12 or 24 weeks (Fig. 5a, c, e). This evaluation was not able to be performed for knit meshes (Phasix, Ventralight ST, and Physiomesh), because they lacked an implant interstitium. This evaluation was also not possible for the biologics after they had remodeled into host tissue.

**Fig. 6:**
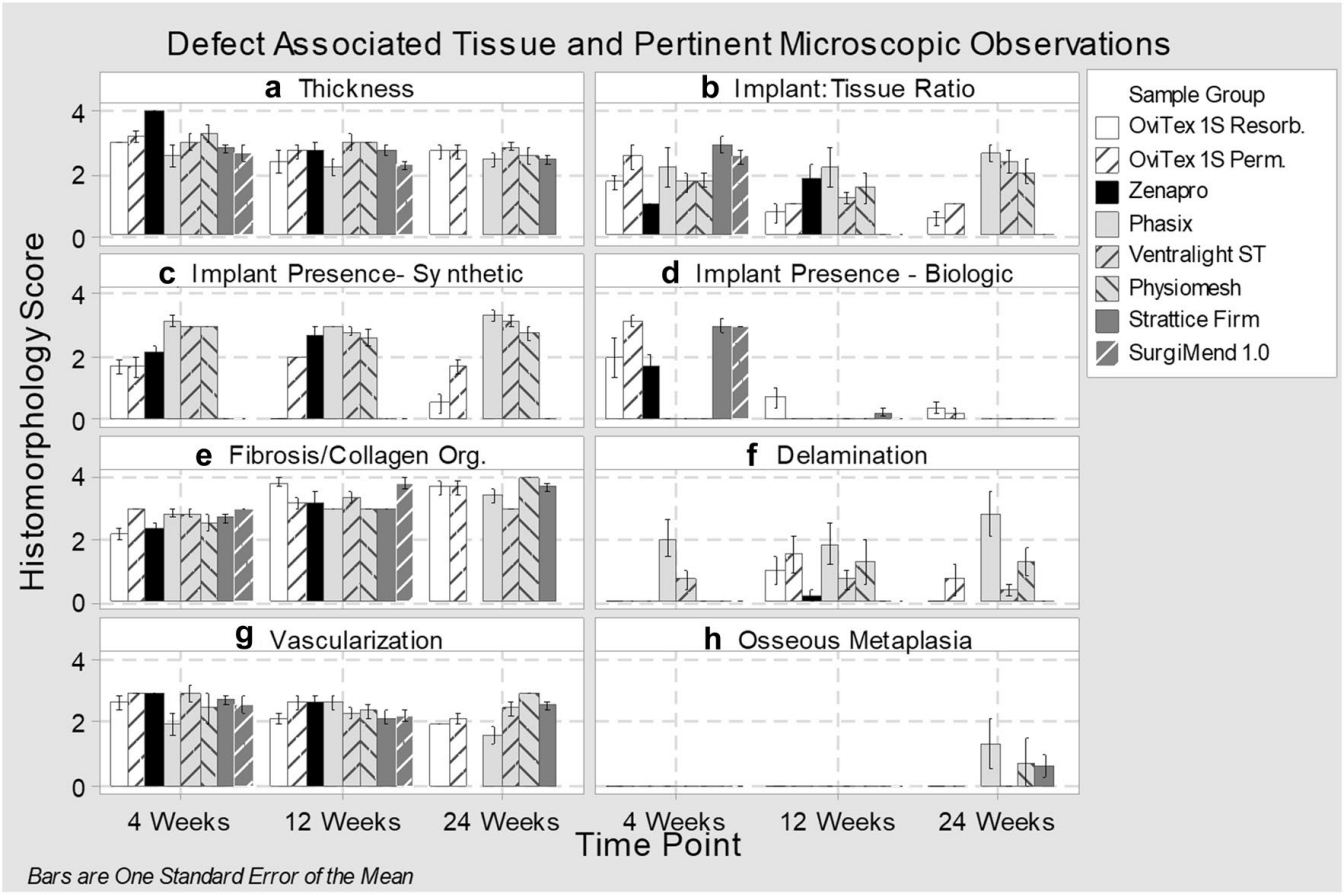
Histomorphological scoring for defect-associated tissue and pertinent microscopic observations at 4, 12, and 24 weeks

Implant-to-tissue ratios at 4 weeks were highest with Strattice Firm, SurgiMend 1.0, and OviTex 1S Permanent (Figs. 2p, s, d, 6b). By 12 weeks, the biologic (Strattice Firm and SurgiMend 1.0) and reinforced biologic implants (both OviTex implants) had remodeled into host tissue which occupied nearly the entire defect area (Fig. 2b, e, q, t). This finding continued at 24 weeks (note SurgiMend 1.0 was not evaluated at this time) (Fig. 6b, d). At both 12 and 24 weeks, the synthetic component of OviTex 1S Permanent was detectable, while the reinforcing component of OviTex 1S Resorbable had resorbed (Fig. 6c). This explains the slightly elevated implant-to-tissue ratio of both OviTex implants in comparison to pure biologic devices. The 24-week remodeled tissue of both OviTex implants and Strattice Firm was comparable to the thickness of the native abdominal wall tissue, with increasing organization of the collagen, consistent with maturation over time (Fig. 6a, e, g).

#### Synthetics

The synthetic implant components of Zenapro, Phasix, Physiomesh, and Ventralight ST were persistently detectable throughout the study, reflecting the abundant content and durable nature of the synthetic polymers in these implants (Figs. 2i, l, o, 6c). Although synthetic implants could not be evaluated for the degree of infiltration, there were host cells and loose connective tissue surrounding the implant fibers; and the organization of an adjacent layer of tissue, which developed asymmetrically on the subcutaneous side of the implant, was analyzed.

Implant-to-tissue ratios among synthetic implants were quantified but confounded by the persistent presence of synthetic meshes occupying a greater portion of the defect thickness over time (Fig. 6b, c). This was further confounded in events where the thickness in relation to the abdominal wall was considerably thin. Furthermore, there were other cases where the mesh bunched up and minimal amounts of deposited collagen were present, resulting in the mesh occupying significant portions of the cross section, and, therefore, a bias in the implant-to-tissue ratio.

Delamination, longitudinal splitting, or clefting along the defect wall repair tissue was also used to qualify the defect tissue area. Delamination was primarily observed in synthetics, in which the mesh was loosely adhered or entirely split from the asymmetrically deposited layer of tissue (Figs. 2g, h, n, o, 6f and Supplementary Fig. 2). Delamination was persistently mild with Phasix, variable but typically mild with Physiomesh, and absent to minimal with Ventralight. Of note, the biologics and reinforced biologics did not develop an asymmetric layer of tissue.

### Implant tissue organization and architecture

Organization (i.e., maturity) of collagenous/fibrous connective tissue was determined by the presence of histologic features including dense lamellar collagen, low cellularity, and predominance of quiescent (fibrocytic) compared to active (fibroblastic) spindle cell morphology (Fig. 6e). At 12 weeks, the organization of tissue spanning the abdominal wall defects was moderately amorphous for Strattice Firm and OviTex 1S Permanent, and markedly lamellar for OviTex 1S Resorbable and SurgiMend 1.0 (Fig. 3q, e, b, t). At 24 weeks, biologics and reinforced biologics had mature lamellar collagen (Fig. 3c, f, r). The tissue adjacent the synthetics was predominantly amorphous, while the area immediately surrounding the mesh fibers was predominantly loose connective tissue with some thin strands of lamellar tissue (Fig. 3i, Supplementary Fig. 2). This finding was one of the more distinct findings between the classes of materials.

Adverse implant findings were limited to mineralization and osseous metaplasia at 24 weeks which was marked/severe in one Phasix implant (also observed at 4 and 12 weeks), moderate in one Strattice Firm implant (also observed at 12 weeks), and moderate in one Physiomesh implant (Supplementary Fig. 3). There were no implant associated cavities/pockets at 24 weeks, regardless of group.

## Discussion

Using a non-human primate model, a new category of hernia repair materials was evaluated in comparison to commercially available meshes. The Old-World primate model was selected, because these primates share greater than 98% of their genes with humans, and, thus, display very similar immune and foreign body responses [15]. The Caribbean Green Vervet monkey has been used extensively to evaluate clinical and immune responses to pathogens, vaccines, and pharmaceuticals, and to predict xenograft biocompatibility for abdominal wall repair [13, 17, 18].

### Materials/properties (source material, ECM, and polymer content)

The implantation of foreign materials stimulates an inflammatory response by the host. Specific characteristics of repair materials used in reconstructive surgery have been linked to different response pathways and help to determine whether the material becomes incorporated into host tissue, encapsulated by scar, or resorbed. For example, certain synthetic repair materials are associated with a potent pro-inflammatory response that results in fibrosis, scarring, and encapsulation [19]. The response to synthetic repair materials is dominated by pro-inflammatory cell phenotypes, chronic inflammation, and increased deposition of scar tissue [20]. The degree of inflammation and foreign body response differs by type of synthetic, and is generally lowest with the lighter weight polypropylene materials and greatest with microporous or heavyweight and polyester materials [20–22].

By contrast, non-crosslinked biologic repair materials that are appropriately decellularized are generally associated with limited foreign body response, reduced chronic inflammation, ingrowth of native tissue, and stimulated implant remodeling [23]. The key differentiator between synthetic and biologic materials is the presence of ECM, which is highly complex and contains a wide variety of bioactive components [8]. These components have demonstrated biological activity, including promotion of angiogenesis, cell adhesion, proliferation, differentiation, and apoptosis; and antimicrobial and chemotactic effects [9].

OviTex test articles were constructed of ECM derived from ovine forestomach (OFM), processed to retain its native structure and tissue ECM components. Characterization of this material has shown the retention of 153 unique matrisome proteins, including 25 collagens, 58 glycoproteins, 12 proteoglycans, 13 ECM affiliated proteins, 20 ECM regulators, and 23 secreted factors [24].

Under normal circumstances, tissue ECM and neighboring cells participate in a continuous feedback, termed ‘dynamic reciprocity’. ECM influences the phenotype and behavior of nearby cells, which in turn remodel the ECM through coordinated degradation and creation of a new matrix [9]. Just like tissue ECM, dECM-based implants function in an identical manner. Initial degradation of biologic repair materials by host cells creates bioactive breakdown products and exposes additional components within the ECM, such as growth factors, that further modulate the host response. This dynamic interaction between host cells and the prosthesis promotes integration of biologic repair material into host tissue.

In this study, the observed limited foreign body response, infiltration by host cells and remodeling, confirmed the benefits of ECM. In comparison to synthetic materials, the reinforced biologics differed with respect to levels of inflammation, specifically the reduction of histiocytic cells. It is believed that the lower quantity of histiocytic cells in OviTex is due not only to the specific ECM, but also to the design and reduced areal density of synthetic materials. Phasix is a mesh with areal density of 182 g/m^2^, which is almost twice that of Marlex (95 g/m^2^), Ventralight ST a mesh at 64 g/m^2^, and Physiomesh a mesh at 30 g/m^2^ [25–28]. By comparison, OviTex 1S Permanent only has less than a fourth the amount of polymer of the Ventralight ST lightweight mesh (15 g/m^2^), and the polymer is embedded in the ECM, which further attenuates any inflammatory response [29]. The observations show that the minimized amount of embedded synthetic reinforcement results in an implant that histologically behaves like a biologic yet maintains its functional structure (i.e., does not stretch).

### Macroscopic architecture and cellular infiltration: channels/pores and permeability

OviTex was purposefully engineered and consists of layers of dECM with channels and pores to promote fluid exchange and allow host cells to penetrate the ECM. Fluid permeability characteristics were optimized and measured. Fluid permeability of OviTex is on average 5 mL/cm^2^/min as compared to the acellular dermal matrices (ADM) (Strattice Firm and SurgiMend 1.0), which were measured to be essentially impermeable (e.g., 0 mL/cm^2^/min) (data unpublished).

At 4 weeks, the OviTex implants had host cells between and within the layers of the implant—this was not seen in Strattice Firm which histologically presented as a dense monoblock of ADM, minimally infiltrated by host cells and covered with a thin superficial layer of fibroblasts. Even though, by 12 weeks, the biologics were infiltrated and were remodeling into host collagen, the early infiltration seen with OviTex “jump started” the transition from implant into integrated host tissue. The earlier infiltration of cells and establishment of a functional vasculature may also reduce the potential for bacterial colonization and, thus, the risk for infection [30].

### Resultant collagen organization

The structure and characteristics of the OviTex products led to more mature and abundant collagen spanning the entire defect area. At 12 weeks, the collagen of OviTex 1S Resorbable was described as lamellar, which matured to a higher degree of organization earlier than the synthetic and, on average, the biologic implants. By 24 weeks, both OviTex implants were remodeled into fully mature tissue. The wavy pattern of dense crimped collagen observed in OviTex is reminiscent of fascia as opposed to the random and amorphous fiber orientation typical of scar. The other biologics also exhibited this trend of increased maturity, notably for SurgiMend 1.0 at 12 weeks and Strattice Firm at 24 weeks. The organization of the collagen adjacent to synthetic meshes was more reminiscent of scar—amorphous, delayed, unorganized, and separated from the mesh by layer of loose connective tissue. No organized collagen was seen within the knitted structure of synthetic meshes.

### Limitations

The predominant limitation of this study is that the model is not per se a biomechanical model of human ventral hernia repair. Although this model does not provide information regarding hernia-related outcomes such as recurrence, the model is effective in allowing for a comparison of a multitude of competitive products in human-equivalent mesh sizes and displays immune/biologic/histologic responses equivalent to those seen in humans. Another limitation was caused by the difficulty in obtaining sufficient supply of some competitive materials, which limited the number of animals that could be studied for certain groups (i.e., SurgiMend 1.0 and Zenapro). Based on the reports in the articles by Sandor 2008 and Xu 2008, it was concluded that the appearance of the implants at the 4-week time point was markedly different from that at the 12 and 24 week time points, which were quite similar in appearance. We, therefore, chose 4 and 12 weeks as the time points to study for these products. Furthermore, this model uses healthy animals. As a result, the quality of the newly formed tissue observed with the materials in this study may differ from the quality of newly formed tissue in patients with various connective tissue disorders.

## Conclusions

The current study shows a favorable response to the reinforced biologics, embroidered with either polypropylene or polyglycolic acid. The test articles were associated with an initial inflammatory response, followed by resolution of inflammation and positive remodeling. By 12 weeks, the test articles were well integrated into host tissues, with little-to-no non-remodeled biologic material remaining. Slight differences in outcomes between OviTex 1S Resorbable versus the OviTex 1S Permanent are due to the minimal inflammatory response to the PP reinforcement, but this did not affect the quality of the final remodeled tissue.

The results also show hernia mesh class differences, and some differences between biologics and reinforced biologics. Synthetic meshes, including the resorbable Phasix, led to an amorphous separated layer of collagen. Biologics and reinforced biologics were more often associated with lamellar collagen, which, in the case of reinforced biologics, generated more rapidly. While these initial in vivo results for the use of reinforced biologics in hernia repair are encouraging, additional clinical validation of the design is on-going via controlled clinical studies out to 24 months [31].

## Electronic supplementary material

Below is the link to the electronic supplementary material.

Supplementary Fig. 1 Gross necropsy and histology of ACell Gentrix Surgical Matrix Plus at 4 Weeks. A) shows implant (*) bunched on one side after pulling away from suture line, B) cross section of implant (*) with bunched up implant, C) histology specimen of largely acellular implant (*) collagen network surrounded by inflammation (TIF 9114 kb)

Supplementary Fig. 2 Synthetic implant delamination of A) Physiomesh 12 weeks and B) Phasix 24 weeks: delamination of amorphous tissue from mesh fibers, separated by moderately thick band of vascular and adipose tissue. C) Phasix 24 weeks: histiocytic response and adipose and loose connective tissue between mesh fibers. Scale bar is 500 µm (TIF 9235 kb)

Supplementary Fig. 3 Adverse implant findings, osseous metaplasia (brackets) of A) Phasix 24 weeks and B) Strattice 24 weeks (TIF 7626 kb)
