# Supplementary figures and images for: In-vivo evaluation of a reinforced ovine biologic: a comparative study to available hernia mesh repair materials
Source: Hernia. 2020 Jan 31;24(6):1293–306. doi: 10.1007/s10029-019-02119-z (PMC7701079; doi:10.1007/s10029-019-02119-z)

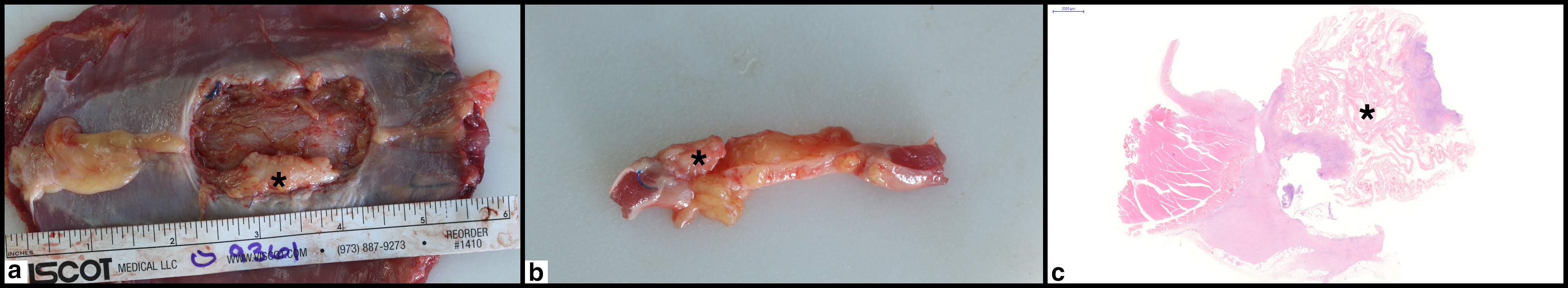

Supplement: Supplementary file 1 — Supplementary Fig. 1 Gross necropsy and histology of ACell Gentrix Surgical Matrix Plus at 4 Weeks. A) shows implant (*) bunched on one side after pulling away from suture line, B) cross section of implant (*) with bunched up implant, C) histology specimen of largely acellular implant (*) collagen network surrounded by inflammation (TIF 9114 kb) [file 10029_2019_2119_MOESM1_ESM.tif]

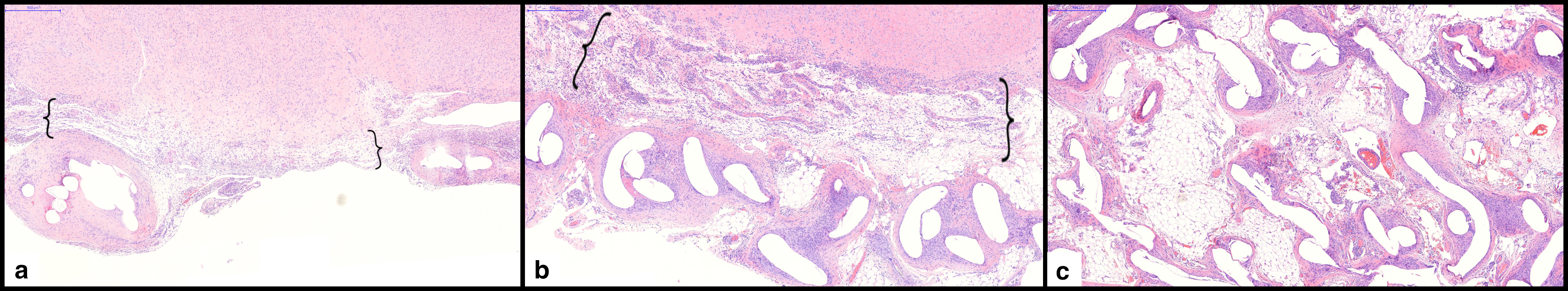

Supplement: Supplementary file 2 — Supplementary Fig. 2 Synthetic implant delamination of A) Physiomesh 12 weeks and B) Phasix 24 weeks: delamination of amorphous tissue from mesh fibers, separated by moderately thick band of vascular and adipose tissue. C) Phasix 24 weeks: histiocytic response and adipose and loose connective tissue between mesh fibers. Scale bar is 500 µm (TIF 9235 kb) [file 10029_2019_2119_MOESM2_ESM.tif]

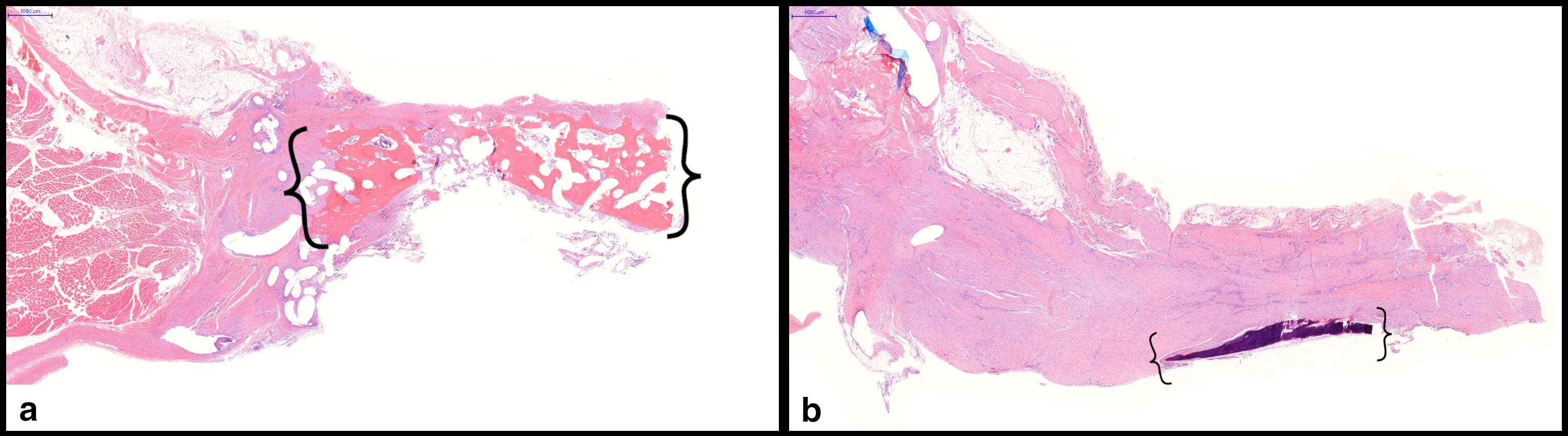

Supplement: Supplementary file 3 — Supplementary Fig. 3 Adverse implant findings, osseous metaplasia (brackets) of A) Phasix 24 weeks and B) Strattice 24 weeks (TIF 7626 kb) [file 10029_2019_2119_MOESM3_ESM.tif]
